# Natural Compounds Regulate Glycolysis in Hypoxic Tumor Microenvironment

**DOI:** 10.1155/2015/354143

**Published:** 2015-01-22

**Authors:** Jian-Li Gao, Ying-Ge Chen

**Affiliations:** Zhejiang Chinese Medical University, No. 548 Binwen Road, Binjiang District, Hangzhou, Zhejiang 310053, China

## Abstract

In the early twentieth century, Otto Heinrich Warburg described an elevated rate of glycolysis occurring in cancer cells, even in the presence of atmospheric oxygen (the Warburg effect). Recently it became a therapeutically interesting strategy and is considered as an emerging hallmark of cancer. Hypoxia inducible factor-1 (HIF-1) is one of the key transcription factors that play major roles in tumor glycolysis and could directly trigger Warburg effect. Thus, how to inhibit HIF-1-depended Warburg effect to assist the cancer therapy is becoming a hot issue in cancer research. In fact, HIF-1 upregulates the glucose transporters (GLUT) and induces the expression of glycolytic enzymes, such as hexokinase, pyruvate kinase, and lactate dehydrogenase. So small molecules of natural origin used as GLUT, hexokinase, or pyruvate kinase isoform M2 inhibitors could represent a major challenge in the field of cancer treatment. These compounds aim to suppress tumor hypoxia induced glycolysis process to suppress the cell energy metabolism or enhance the susceptibility of tumor cells to radio- and chemotherapy. In this review, we highlight the role of natural compounds in regulating tumor glycolysis, with a main focus on the glycolysis under hypoxic tumor microenvironment.

## 1. Warburg Effect, Glycolysis, and Tumor Hypoxia

Cells regulate glucose metabolism based on their growth and differentiation status, as well as the molecular-oxygen deficiency. The discrepancy between the rapid rate of tumor growth and the capacity of existing blood vessels to supply oxygen and nutrients makes the adaptation to hypoxia environment become the basis for the survival and growth of tumor cells. In the process of cancer metabolic reprogramming, tumor cells adapt to hypoxia through enhancing glycolysis [1]. Therefore, the induction of the glycolysis is essential for cancer cell survival under hypoxic microenvironment, and the process of tumor growth and metastasis were promoted by hypoxic or acidic extracellular microenvironment.

Glycolysis is the metabolic process in which glucose is converted into pyruvate. In normal cells, glycolysis is prioritized only when oxygen supply is limited. When oxygen is present, pyruvate then enters the mitochondrial tricarboxylic acid (TCA) cycle to be fully oxidized to CO_2_ (oxidative phosphorylation). However, when the function of mitochondria was damaged or under hypoxic conditions, pyruvate is instead converted into lactate in anaerobic glycolysis [2]. In contrast with normal cell, cancer cells preferentially use glycolysis even in the abundance of oxygen. Therefore, tumor glycolysis is often called “aerobic glycolysis,” or the Warburg effect to distinguish from the normal glycolysis. Tumor glycolysis provides energy for rapid tumor growth and promotes cancer metastasis.

**Figure 1:**
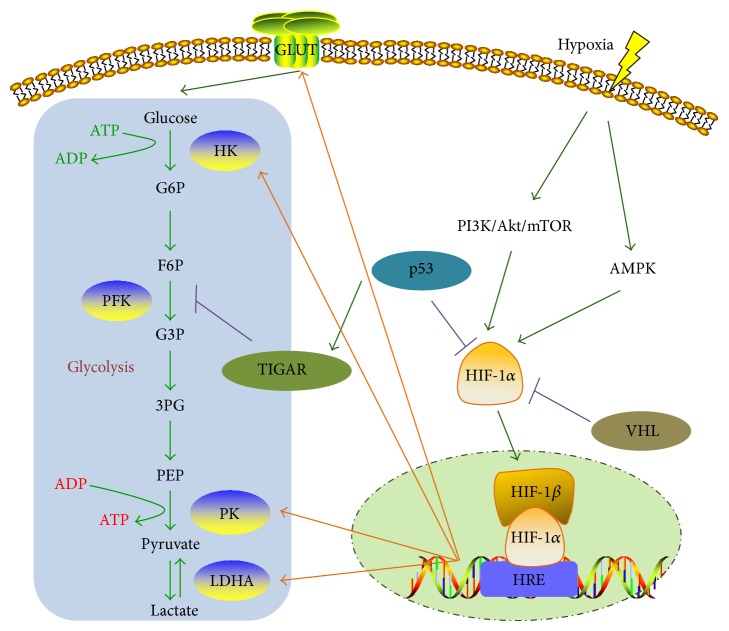
Signaling pathways and key factors involved in hypoxic induced Warburg effect. GLUT: glucose transporter; G6P: glucose-6-phosphate; HK: hexokinase; F6P: fructose-6-phosphate; PFK: phosphofructokinase; G3P: glyceraldehyde-3-phosphate; 3PG: 3-phosphoglycerate; PEP: phosphoenolpyruvate; PK: pyruvate kinase; PKM2: pyruvate kinase isoform M2; LDHA: lactate dehydrogenase; HIF: hypoxia-inducible factor; AMPK: adenosine 5′-monophosphate- (AMP-) activated protein kinase; PI3K: phosphoinositide-3-kinase; mTOR: mammalian target of rapamycin; HRE: hypoxia response element; VHL: Von Hippel-Lindau; TIGAR: TP53-induced glycolysis and apoptosis regulator.

Hypoxia inducible factor-1 (HIF-1) is a key transcription factor that plays major roles in this metabolic reprogramming (Figure 1). In agreement with the results from invertebrate models, it is now known that adenosine 5′-monophosphate- (AMP-) activated protein kinase (AMPK), phosphoinositide-3-kinase (PI3K)/Akt, and extracellular regulated protein kinase (ERK) are important signaling pathways to promote cancer glucose metabolic process. In contrast, major tumor suppressors such as P53 and von Hippel-Lindau (VHL) antagonize those changes and keep cellular metabolism in check. HIF-1 subsequently upregulates the glucose transporters, especially glucose transporter 1 (GLUT1) and GLUT4, and induces the expression of glycolytic enzymes, such as hexokinase (HK), pyruvate kinase (PK), and lactate dehydrogenase (LDH-A).

Recently, accumulating evidence concerns natural compounds and cancer glucose metabolism. These compounds display antitumor activity to a range of human cancer cells through adapting the glucose absorption/metabolism. In comparison with synthetic compounds, natural molecules have wide range of sources, diversiform structures, multiple targets, and diversified pharmacological potential, which provide a considerable source for glycolysis inhibitors. In this review, we discuss the role of natural compounds in the regulation of aerobic glycolysis which is induced by HIF-1 and their influence on tumor growth and metastasis.

## 2. Natural Compounds as Regulators of HIF-1 Induced Warburg Effect

### 2.1. Inhibitors Focus on the Glycolysis-Related Factors

#### 2.1.1. Glucose Transporters

Glucose transporters and other dehydrogenates were closely related to glycolysis. Many natural compounds most likely affect expression of glucose transporters (especially GLUT1 and GLUT4) indirectly, rather controlling upstream modulatory mechanisms. Flavones, polyphenols, and alkaloids are interesting bioactive anticancer molecules isolated from plants, as several of them have been repeatedly reported to control glucose transporter activity in different cancer cell models (Table 1). Fisetin, myricetin, quercetin, apigenin, genistein, cyanidin, daidzein, hesperetin, naringenin, and catechin are well-known inhibitors of glucose uptake in human U937 cells [4]. As a matter of fact, comparative studies indicated that these compounds do not exhibit the same mode of action as they bind different domains of GLUT1. Genistein binds the transporter on the external face whereas quercetin interacts with the internal face [7].

The report of Vaughan et al. indicated that aerobic glycolysis can be directly induced by an inflammatory microenvironment independent of additional genetic mutations and signals from adjacent cells, and curcumin could reverse this effect [6]. Another natural compound, 4-O-methyl alpinumisoflavone, isolated from* Lonchocarpus glabrescens*, could inhibit HIF-1 activation and hypoxic induction of HIF-1 target genes (CDKN1A, GLUT-1, and vascular endothelial growth factor (VEGF)) [9].

Besides, annonaceous acetogenins, long chained fatty acid derivatives extracted from* Annona muricata* (Graviola), have recently shown multiple anticancer activities on pancreatic cancer cell models and mouse xenograft models. Torres et al. highlighted the ability of this compound to inhibit glucose uptake, and it has strong ability to reduce the expression levels of GLUT1 and GLUT4, HKII, and LDH-A [8].

#### 2.1.2. Hexokinase

In the present time, as a HK inhibitor, lonidamine has become new drug that interferes with mitochondrial functions, thereby inhibiting cellular oxygen consumption and energy metabolism in both normal and neoplastic cells. 2-Deoxyglucose, another HK inhibitor, was in its Phase I/II trial stage for treatment of advanced cancer and hormone refractory prostate cancer [17]. Some natural compounds have been described as promoting the detachment of HK from mitochondria. Deng et al. reported that a novel small-molecular compound neoalbaconol (NA), isolated from the fungus,* Albatrellus confluens*, could target 3-phosphoinositide-dependent protein kinase 1 (PDK1) and inhibit its downstream phosphoinositide-3 kinase (PI3-K)/Akt-HK2 pathway and then resulted in energy depletion [11]. Prosapogenin A, a saponin from Chinese herb* Veratrum nigrum* L., could inhibit cell growth and promote cell apoptosis of MCF7 via inhibition of signal transducer and activator of transcription 3 (STAT3) and glycometabolism-related gene, namely, GLUT1, HK, and PFKL [13]. Methyl jasmonate, a plant stress hormone produced by many plants including rosemary, olive, or ginger, binds to HK and perturbs its association with the voltage-dependent anion channel (VDAC), which leads to overall energetic impairment, and promotes the release of cytochrome c from mitochondria, triggering apoptosis in cancer cells [10].

#### 2.1.3. Pyruvate Kinase M2

As an isozyme of pyruvate kinase that is specifically expressed in cancer cells, PKM2 plays an important role in the metabolism of cancer cells. The increase of tetrameric versus dimeric PKM2 isoform ratio abrogates the Warburg effect and may reactivate oxidative phosphorylation [18]. A report from Liu's group identified that oleanolic acid is an antitumor compound that suppresses aerobic glycolysis in cancer cells and there is potential that PKM2 may be developed as an important target in aerobic glycolysis pathway for developing novel anticancer agents [12]. Shikonin, a small-molecule natural product which inhibits the activity of PKM2, has a synergistic effect with Taxol; this effect was involved in its inhibition of ERK, Akt, and p70S6 kinases [15]. High-throughput screenings based on an enzymatic LDH assay were used to identify PKM2 inhibitor from a compound library of the Food and Drug Administration (FDA) [19]. Accordingly, three potential chemical structures, including thiazolidinediones and natural compounds belonging to the group of naphthoquinones: shikonin, alkannin, and their derivatives (extracted from different plants including* Arnebia sp.* and* Alkanna tinctoria*), have been recently shown as the most potent and specific inhibitors of PKM2 [3].

#### 2.1.4. Lactate Dehydrogenase A

In recent years, LDH-A is emerging as a novel therapeutic target in inhibiting cancer aerobic glycolysis. As an important factor in nicotinamide adenine dinucleotide (NAD^+^) regeneration, LDH-A was overexpressed in various types of cancer including renal, breast, gastric, and nasopharyngeal cancer [20, 21]. Inhibition of LDH-A might lead to energy production blockade in cancer cells, such as the reactive oxygen species (ROS) burst, mitochondrial pathway apoptosis, and limited tumorigenic abilities [22, 23]. It has also been recently shown that the* Spatholobus suberectus* aqueous extract has LDH-A inhibiting activity and the possibility to consider epigallocatechin as a lead compound to develop LDH-A inhibitors [16]. But other natural compounds, such as furanodiene and maslinic acid, could increase the LDH release in cancer cells by inducing cancer cell injury [24, 25]. Hence, the change of LDH-A level (both enhancing and decreasing) could prevent cancer proliferation by inhibition metabolism or inducing cell damage.

#### 2.1.5. Glyceraldehyde-3-Phosphate Dehydrogenase

Glyceraldehyde-3-phosphate dehydrogenase (GAPDH) is another key glycolytic enzyme which may play multiple noncanonical functions implicated in cell growth and survival by hypoxic-independence pathway. Saframycin A, a bacterial product of fermentation, may form a nuclear ternary complex with GAPDH and DNA and consequently exhibits antiproliferative properties in both adherent and nonadherent cancer cell models [14].

### 2.2. Inhibitors Focus on the Expression and Transcriptional Activation of HIF-1*α*, VHL, and P53

As the key transcription factor of glycolytic process, many tumor suppressors with emerging role in regulation of aerobic glycolysis may control glycolytic genes' expression through HIF-1*α* regulation. For example, WW domain-containing oxidoreductase (WWOX), as a modulator of cancer metabolism, via its first WW domain, physically interacts with HIF1*α* and modulates its levels and transactivation function. Consequently, WWOX downregulated GLUT1 levels and inhibited glycolysis in breast cancer samples [26]. Besides, P53 and VHL are also recognized as metabolic tumor suppressors via HIF-1*α* modulation. Recent studies have revealed that tumor suppressor P53 represses glycolysis under normoxia as a novel mechanism for tumor suppression [27]. Research also indicated that VHL inactivation accelerated hepatic glucose storage through the upregulation of IGF-IR and GLUT1 and that IGF-IR was a key regulator in VHL-deficient hepatocytes [28].

#### 2.2.1. HIF-1*α*

Hypoxia can regulate erythropoietin, tyrosine hydroxylase enzyme, glucose transporter 1, glycolytic enzymes, and VEGF and a series of hypoxia induced HIF-1 gene expressions, resulting in tumor proliferation, invasion, migration, and adhesion, is constantly an important cause of malignant tumor. There is no doubt that HIF-1 is a central molecule in the control of the expression of glucose transporters and key glycolytic enzymes as well. Because HIF-1*α* is overexpressed in different human cancers and their metastases, the inhibition of the HIF-1 pathway represents a promising approach in cancer therapy.

In the past few years, many studies tried to identify natural compounds able to interfere with or inhibit the HIF-1 activity [29]. Several potential novel HIF-1 inhibitors were discovered, such as triptolide [30], emetine, klugine, isocephaeline [31], manassantin B, 4-o-demethylmanassantin [32], alpinumisoflavone and 4-O-methyl alpinumisoflavone [9], kaempferol [33], rhein [34], pseudolaric acid B [35], piceatannol [36], beturetol, and isosakuranetin [37]. These compounds inhibit hypoxia-induced HIF-1 activation; besides, they may affect the expression of HIF-1 and HIF-1 target genes including GLUT1.

Similarly, other research groups have identified that the cinnamic acid derivatives baccharin and drupanin, extracted from the Brazilian green propolis, could inhibit the expression of HIF-1 and its target genes (GLUT1, HKII, and VEGF) as inhibitors of HIF-1-dependent luciferase activity [37]. Another compounds, brucine, could suppress HIF-1-dependent luciferase activity in HepG2 cells and show a dose-dependent inhibition effect in the lung metastasis of H22 ascitic hepatoma cells in tumor-bearing mice. The inhibition of the HIF-1 pathway is implicated in the antimetastasis activity of brucine [38]. Thyrsiferol, marine red algal metabolite, was found to inhibit HIF-1 activation in T47D human breast tumor cells and suppressed HIF-1 target genes (VEGF, GLUT-1) at the mRNA level [39].

#### 2.2.2. VHL

Von Hippel-Lindau (VHL) is one of the most important tumor suppressor genes and negative regulator of hypoxic signaling pathway. Nepal et al. show that bavachinin inhibited increases in HIF-1*α* activity in human KB carcinoma (HeLa cell derivative) and human HOS osteosarcoma cells under hypoxia in a concentration-dependent manner, probably by enhancing the interaction between VHL and HIF-1*α* [40]. On the other hand, although the loss of VHL enables the survival and proliferation of cells, it is also expected to introduce vulnerabilities which may be unrelated to HIF and exploited for therapeutics discovery. Woldemichael et al. found carminomycin I as an effective inhibitor of VHL defective (VHL^−/−^) clear cell renal cell carcinoma (CCRCC) cell proliferation and the P-gp mediated localization of carminomycin I in CCRCC cells [41].

#### 2.2.3. P53

P53 is a tumor suppressor, induces cell-cycle arrest and cell death after DNA damage, and thus contributes to the maintenance of genomic stability. In addition to this tumor suppressor function for prooncogenic cells, P53 also negatively regulates glycolysis through activation of TIGAR (TP53-induced glycolysis regulator, an inhibitor of the fructose-2,6-bisphosphate) [39]. The findings of the various studies concerning natural compounds indicate that the anticancer mechanisms of several compounds were involved in the expression and activation of P53. Lin et al. found that caffeine reduced p53*α* expression and induced the expression of p53*β*, which contains an alternatively spliced p53 C-terminus, via the alternative splicing of the target genes of serine/arginine-rich splicing factor 3 (SRSF3). And caffeine also induced the alternative splicing of other SRSF3 target genes, such as GLUT1, HIF-1*α*, and HIF-2*α* [42].

### 2.3. Inhibitors Focus on the Upstream Signaling Pathway of Hypoxia Induced Glycolysis

The metabolic signature of cancer cells correlates with defects in several signaling pathways. Among them, phosphoinositide-3-kinase (PI3K) and AMPK are essentially implicated.

#### 2.3.1. PI3K Pathway

PI3K is an important molecular signal transduction in cells. PI3K can be growth factors, cytokines, hormones, and other extracellular signals activator and also affect cellular functions, such as glucose metabolism. As mentioned above, neoalbaconol (NA) reduced the consumption of glucose and ATP generation by targeting 3-phosphoinositide-dependent protein kinase 1 (PDK1) and inhibiting its downstream PI3-K/Akt-HK2 pathway [11]. The serine/threonine mammalian target of rapamycin (mTOR) is a critical component of an adaptive system that senses the availability of a variety of nutrients and growth factors in the microenvironment and appears as a crucial controller of metabolic homeostasis. Oleanolic acid has been discovered as a new class of glucose metabolism inhibitors; oleanolic acid's effect on PKM2/PKM1 switch is found to be involved in the inactivation of mTOR signaling [12].

Wogonin could also be a good candidate for the development of new multidrug resistance (MDR) reversal agent and its reversal mechanism probably is due to the suppression of HIF-1*α* expression via inhibiting PI3K/Akt signaling pathway. Wogonin suppressed the expression of glycolysis-related proteins (HKII, pyruvate dehydrogenase kinase 1 (PDHK1), and LDH-A), glucose uptake, and lactate generation in a dose-dependent manner. Further, wogonin could downregulate HIF-1*α* expression and glycolysis through inhibiting PI3K/Akt signaling pathway both* in vitro *and* in vivo*, which might be the mechanism of reversal resistance of wogonin [43, 44].

#### 2.3.2. AMPK Pathway

The AMP-activated protein kinase (AMPK) is considered as a key checkpoint to ensure energy balance in both cells and organisms. It negatively regulates aerobic glycolysis in cancer cells and suppresses tumor growth* in vivo* [45]. It was found that AMPK supports tumor glucose metabolism in part through positive regulation of glycolysis and the nonoxidative pentose phosphate cycle [46]. Molecular analysis indicated that hypoxia upregulated the key proteins glucose transport and glycolysis, GLUT1, and 6-phosphofructo-2-kinase and that these changes were induced by HIF-1 upregulation and/or AMPK activation [47].

Liu's research found that AMPK activation is required for the antitumor activity of oleanolic acid on cancer cells. Oleanolic acid was found to activate AMPK, the master regulator of metabolism, in prostate cancer cell line PC-3 and breast cancer cell line MCF-7. Aerobic glycolysis was inhibited in cancer cells treated with oleanolic acid, in an AMPK activation-dependent manner [48]. WZB117 is a prototype for further development of anticancer therapeutics targeting GLUT 1-mediated glucose transport and glucose metabolism. WZB117 could reduce the levels of GLUT 1 protein, intracellular ATP, and glycolytic enzymes. All these changes were followed by increase in ATP sensing enzyme AMPK and declines in cyclin E2 as well as phosphorylated retinoblastoma, resulting in cell-cycle arrest, senescence, and necrosis [49]. Cannabinoids, a class of bioactive lipids that have a range of interesting activities, reduce the growth of tumours such as glioma, breast cancer, prostate cancer, and colon cancer. Studies on cannabinoids indicated that cannabinoids could inhibit activity of PKM2, further downregulating glycolysis, and glutamine uptake by AMPK-dependent pathway [50].

## 3. Conclusions

Discovery of druggable mediators of cancer glucose metabolism becomes an increasingly interesting research field. In comparison with synthetic compounds, natural molecules exert multiple advantages due to their large-scale structure and diversity targets. Currently, researches have been engaged in antitumor metabolism lead compound discovery by targeting the key targets or pathways involved in the glycolysis. Firstly, compounds directly reducing the glucose uptake could be the candidate for cancer glucose metabolic inhibitor. Secondly, any compounds able to inhibit the expression or activity of glycolytic enzymes could also inhibit the tumor glycolysis. Thirdly, targeting of HIF-1*α* and hypoxic-related factors should impair cancer cell survival either by attenuating tumor glucose metabolic processes or by inhibiting VEGF induced prosurvival and angiogenesis pathways. Finally, regulators focused on upstream pathways of HIF-1*α* and glycolysis, especially PI3K and AMPK pathways, could also be a source of tumor metabolic inhibitor or energy restriction mimetic agents (ERMAs).

Tumor cells reprogram their glucose metabolism to rely largely on glycolysis for their energy need, even in the presence of adequate oxygen. In this view, putative modulators of cancer cell metabolism might represent a new effective class of single treatments or chemoadjuvants in cancer therapy. The emerging interplay between cancer cell metabolism and altered gene expression in cancer suggests that many of the anticancer activities ascribed to natural compounds are in fact the consequence of preventing deregulated cancer cell metabolism and growing evidence confirms this hypothesis.

Recently, accumulating evidence supports that noncoding RNAs (microRNA and LncRNA) participate in many physiological processes by modulating gene expression at the epigenetic, transcriptional, and posttranscriptional levels. At the light of this new vision, it will be important to better understand this specific area on future research and it is also essential for discovering the small molecular compounds which could affect the function or level of noncoding RNAs, such as LncRNA-UCA1 and miR143 [51].

## Figures and Tables

**Table 1:** Natural compounds interfere with glycolysis signaling pathway and function.

Compound/extract name	Chemical class	Effects on glycolysis and potential mechanisms of action	References
Alkannin	Naphthoquinone	Inhibits the activity of PKM2	[3]
Apigenin	Flavones	Inhibits glucose uptake in U937 and MC3T3-G2/PA6 cells and inhibits activation of Akt and translocation of GLUT4	[4, 5]
Catechin	Flavanol	Inhibits glucose uptake in U937	[4]
Cyanidin	Flavanol	Inhibits glucose uptake in U937	[4]
Curcumin	Polyphenol	Inhibits inflammatory-induced glycolysis	[6]
Daidzein	Isoflavone	Inhibits glucose uptake in U937	[4]
(−)-Epigallocatechin gallate	Flavanol	Inhibits insulin-stimulated glucose uptake in mouse MC3T3-G2/PA6 cells	[5]
Fisetin	Flavonol	Inhibits glucose uptake in U937 and MC3T3-G2/PA6 cells	[4, 5]
Genistein	Isoflavone	Inhibits glucose uptake in U937 and binds on the external surface of GLUT1	[4, 7]
Graviola extract		Inhibits glucose uptake and strongly reduces the GLUT1, GLUT4, HKII, and LDH-A expression	[8]
Hesperetin	Flavanone	Inhibits glucose uptake in human myelocytic U937	[4]
Kaempferol	Flavonol	Inhibits insulin-stimulated glucose uptake in mouse MC3T3-G2/PA6 cells and inhibits activation of Akt and translocation of GLUT4	[5]
Luteolin	Flavones	Inhibits insulin-stimulated glucose uptake in mouse MC3T3-G2/PA6 cells, inhibits insulin-stimulated phosphorylation of IR-*β*, and inhibits activation of Akt and translocation of GLUT4	[5]
4-O-Methyl alpinumisoflavone	Isoflavone	HIF-1 inhibitor; inhibits HIF-1 target genes GLUT-1	[9]
Methyl jasmonate	Methyl ester	Detaches hexokinase from the mitochondria	[10]
Myricetin	Flavonol	Inhibits glucose uptake in human U937 cells	[4]
Naringenin	Flavanone	Inhibits glucose uptake in human U937 cells	[4]
Neoalbaconol	Sesquiterpenes	Inhibits PI3-K/Akt-HK2 pathway	[11]
Oleanolic acid	Organic acid	Induces PKM2/PKM1 switch and suppresses aerobic glycolysis	[12]
Prosapogenin A	Saponin	Inhibition of STAT3 and GLUT1, HK, and liver-type subunit of phosphofructokinase (PFKL)	[13]
Quercetin	Flavonol	Inhibits glucose uptake in U937 and MC3T3-G2/PA6 cells, inhibits activation of Akt and translocation of GLUT4, and binds on the internal side of GLUT1	[4, 5, 7]
Saframycin A	Alkaloid	Forms a nuclear ternary complex with GAPDH and DNA	[14]
Shikonin	Naphthoquinone	Inhibits the activity of PKM2	[15]
Silybin	Flavanonol	Inhibits insulin-stimulated glucose uptake in mouse MC3T3-G2/PA6 cells	[5]
*Spatholobus suberectus* aqueous extract		Inhibiting cancer LDH-A activity	[16]
Theaflavins	Flavanol	Inhibit insulin-stimulated glucose uptake in mouse MC3T3-G2/PA6 cells	[5]

## Abbreviations

AMPK: Adenosine 5′-monophosphate- (AMP-) activated protein kinase
ERK: Extracellular regulated protein kinase
GAPDH: Glyceraldehyde-3-phosphate dehydrogenase
GLUT: Glucose transporter
HIF: Hypoxia-inducible factor
HK: Hexokinase
HRE: Hypoxia response element
IGF-IR: Insulin-like growth factor I receptor
IR-*β*: Insulin receptor-*β* subunit
LDH-A: Lactate dehydrogenase
mTOR: Mammalian target of rapamycin
NAD^+^: Nicotinamide adenine dinucleotide
PDHK: Pyruvate dehydrogenase kinase
PFKL: Liver-type subunit of phosphofructokinase
PI3K: Phosphoinositide-3-kinase
PKM2: Pyruvate kinase isoform M2
ROS: Reactive oxygen species
SRSF3: Serine/arginine-rich splicing factor 3
STAT3: Signal transducer and activator of transcription 3
TIGAR: TP53-induced glycolysis and apoptosis regulator
VDAC: Voltage-dependent anion channel
VEGF: Vascular endothelial growth factor
VHL: Von Hippel-Lindau
WWOX: WW domain-containing oxidoreductase.

## References

[1] Hanahan D., Weinberg R. A. (2011). Hallmarks of cancer: the next generation. *Cell*.

[2] Heiden M. G. V., Cantley L. C., Thompson C. B. (2009). Understanding the warburg effect: the metabolic requirements of cell proliferation. *Science*.

[3] Chen J., Xie J., Jiang Z., Wang B., Wang Y., Hu X. (2011). Shikonin and its analogs inhibit cancer cell glycolysis by targeting tumor pyruvate kinase-M2. *Oncogene*.

[4] Park J. B. (1999). Flavonoids are potential inhibitors of glucose uptake in U937 cells. *Biochemical and Biophysical Research Communications*.

[5] Nomura M., Takahashi T., Nagata N. (2008). Inhibitory mechanisms of flavonoids on insulin-stimulated glucose uptake in MC3T3-G2/PA6 adipose cells. *Biological and Pharmaceutical Bulletin*.

[6] Vaughan R. A., Garcia-Smith R., Dorsey J., Griffith J. K., Bisoffi M., Trujillo K. A. (2013). Tumor necrosis factor alpha induces Warburg-like metabolism and is reversed by anti-inflammatory curcumin in breast epithelial cells. *International Journal of Cancer*.

[7] Pérez A., Ojeda P., Ojeda L. (2011). Hexose transporter GLUT1 harbors several distinct regulatory binding sites for flavones and tyrphostins. *Biochemistry*.

[8] Torres M. P., Rachagani S., Purohit V. (2012). Graviola: a novel promising natural-derived drug that inhibits tumorigenicity and metastasis of pancreatic cancer cells in vitro and in vivo through altering cell metabolism. *Cancer Letters*.

[9] Liu Y., Venna C. K., Morgan J. B. (2009). Methylalpinumisoflavone inhibits hypoxia-inducible factor-1 (HIF-1) activation by simultaneously targeting multiple pathways. *Journal of Biological Chemistry*.

[10] Cohen S., Flescher E. (2009). Methyl jasmonate: a plant stress hormone as an anti-cancer drug. *Phytochemistry*.

[11] Deng Q., Yu X., Xiao L. (2013). Neoalbaconol induces energy depletion and multiple cell death in cancer cells by targeting PDK1-PI3-K/Akt signaling pathway. *Cell Death and Disease*.

[12] Liu J., Wu N., Ma L. (2014). Oleanolic acid suppresses aerobic glycolysis in cancer cells by switching pyruvate kinase type M isoforms. *PLoS ONE*.

[13] Wang T.-X., Shi X.-Y., Cong Y., Zhang Z.-Q., Liu Y.-H. (2013). Prosapogenin A inhibits cell growth of MCF7 via downregulating STAT3 and glycometabolism-related gene. *Yao Xue Xue Bao*.

[14] Xing C., LaPorte J. R., Barbay J. K., Myers A. G. (2004). Identification of GAPDH as a protein target of the saframycin antiproliferative agents. *Proceedings of the National Academy of Sciences of the United States of America*.

[15] Li W., Liu J., Jackson K., Shi R., Zhao Y. (2014). Sensitizing the therapeutic efficacy of taxol with shikonin in human breast cancer cells. *PLoS ONE*.

[16] Wang Z., Wang D., Han S. (2013). Bioactivity-guided identification and cell signaling technology to delineate the lactate dehydrogenase A inhibition effects of *Spatholobus suberectus* on breast cancer. *PLoS ONE*.

[17] Pathania D., Millard M., Neamati N. (2009). Opportunities in discovery and delivery of anticancer drugs targeting mitochondria and cancer cell metabolism. *Advanced Drug Delivery Reviews*.

[18] Anastasiou D., Yu Y., Israelsen W. J. (2012). Pyruvate kinase M2 activators promote tetramer formation and suppress tumorigenesis. *Nature Chemical Biology*.

[19] Vander Heiden M. G., Christofk H. R., Schuman E. (2010). Identification of small molecule inhibitors of pyruvate kinase M2. *Biochemical Pharmacology*.

[20] Kolev Y., Uetake H., Takagi Y., Sugihara K. (2008). Lactate dehydrogenase-5 (LDH-5) expression in human gastric cancer: association with hypoxia-inducible factor (HIF-1*α*) pathway, angiogenic factors production and poor prognosis. *Annals of Surgical Oncology*.

[21] Xie H., Valera V. A., Merino M. J. (2009). LDH-A inhibition, a therapeutic strategy for treatment of hereditary leiomyomatosis and renal cell cancer. *Molecular Cancer Therapeutics*.

[22] Wang Z.-Y., Loo T. Y., Shen J.-G. (2012). LDH-A silencing suppresses breast cancer tumorigenicity through induction of oxidative stress mediated mitochondrial pathway apoptosis. *Breast Cancer Research and Treatment*.

[23] Fantin V. R., St-Pierre J., Leder P. (2006). Attenuation of LDH-A expression uncovers a link between glycolysis, mitochondrial physiology, and tumor maintenance. *Cancer Cell*.

[24] Zhong Z., Dang Y., Yuan X. (2012). Furanodiene, a natural product, inhibits breast cancer growth both in vitro and in vivo. *Cellular Physiology and Biochemistry*.

[25] Qian Y., Guan T., Tang X. (2011). Maslinic acid, a natural triterpenoid compound from *Olea europaea*, protects cortical neurons against oxygen-glucose deprivation-induced injury. *European Journal of Pharmacology*.

[26] Abu-Remaileh M., Aqeilan R. I. (2014). Tumor suppressor WWOX regulates glucose metabolism via HIF1*α* modulation. *Cell Death and Differentiation*.

[27] Zhang C., Liu J., Liang Y. (2013). Tumour-associated mutant p53 drives the Warburg effect. *Nature Communications*.

[28] Kurabayashi A., Kakinuma Y., Morita T., Inoue K., Sato T., Furihata M. (2013). Conditional VHL gene deletion causes hypoglycemic death associated with disproportionately increased glucose uptake by hepatocytes through an upregulated IGF-I receptor. *PLoS ONE*.

[29] Nagle D. G., Zhou Y.-D. (2009). Marine natural products as inhibitors of hypoxic signaling in tumors. *Phytochemistry Reviews*.

[30] Chen F., Liu Y., Wang S. (2013). Triptolide, a Chinese herbal extract, enhances drug sensitivity of resistant myeloid leukemia cell lines through downregulation of HIF-1*α* and Nrf2. *Pharmacogenomics*.

[31] Zhou Y.-D., Kim Y.-P., Mohammed K. A. (2005). Terpenoid tetrahydroisoquinoline alkaloids emetine, klugine, and isocephaeline inhibit the activation of hypoxia-inducible factor-1 in breast tumor cells. *Journal of Natural Products*.

[32] Hodges T. W., Hossain C. F., Kim Y.-P., Zhou Y.-D., Nagle D. G. (2004). Molecular-targeted antitumor agents: The Saururus cernuus dineolignans manassantin B and 4-O-demethylmanassantin B are potent inhibitors of hypoxia-activated HIF-1. *Journal of Natural Products*.

[33] Mylonis I., Lakka A., Tsakalof A., Simos G. (2010). The dietary flavonoid kaempferol effectively inhibits HIF-1 activity and hepatoma cancer cell viability under hypoxic conditions. *Biochemical and Biophysical Research Communications*.

[34] Fernand V. E., Losso J. N., Truax R. E. (2011). Rhein inhibits angiogenesis and the viability of hormone-dependent and -independent cancer cells under normoxic or hypoxic conditions *in vitro*. *Chemico-Biological Interactions*.

[35] Li M.-H., Miao Z.-H., Tan W.-F. (2004). Pseudolaric acid B inhibits angiogenesis and reduces hypoxia-inducible factor 1*α* by promoting proteasome-mediated degradation. *Clinical Cancer Research*.

[36] Yum S., Doh H.-J., Hong S. (2013). Piceatannol, a hydroxystilbene natural product, stabilizes HIF-1*α* protein by inhibiting HIF prolyl hydroxylase. *European Journal of Pharmacology*.

[37] Hattori H., Okuda K., Murase T. (2011). Isolation, identification, and biological evaluation of HIF-1-modulating compounds from Brazilian green propolis. *Bioorganic and Medicinal Chemistry*.

[38] Shu G., Mi X., Cai J. (2013). Brucine, an alkaloid from seeds of *Strychnos nux-vomica* Linn., represses hepatocellular carcinoma cell migration and metastasis: the role of hypoxia inducible factor 1 pathway. *Toxicology Letters*.

[39] Madan E., Gogna R., Bhatt M., Pati U., Kuppusamy P., Mahdi A. A. (2011). Regulation of glucose metabolism by p53: emerging new roles for the tumor suppressor. *Oncotarget*.

[40] Nepal M., Jung Choi H., Choi B.-Y. (2012). Anti-angiogenic and anti-tumor activity of Bavachinin by targeting hypoxia-inducible factor-1*α*. *European Journal of Pharmacology*.

[41] Woldemichael G. M., Turbyville T. J., Linehan W. M., McMahon J. B. (2011). Carminomycin I is an apoptosis inducer that targets the golgi complex in clear cell renal carcinoma cells. *Cancer Research*.

[42] Lin W.-S., Lu G.-Y., Huang S.-M., Liu S.-T., Liu P.-Y., Chou W.-Y. (2014). Caffeine induces tumor cytotoxicity via the regulation of alternative splicing in subsets of cancer-associated genes. *The International Journal of Biochemistry & Cell Biology*.

[43] Wang H., Zhao L., Zhu L.-T. (2014). Wogonin reverses hypoxia resistance of human colon cancer HCT116 cells via downregulation of HIF-1*α* and glycolysis, by inhibiting PI3K/Akt signaling pathway. *Molecular Carcinogenesis*.

[44] Zhao K., Song X., Huang Y. (2014). Wogonin inhibits LPS-induced tumor angiogenesis via suppressing PI3K/Akt/NF-*κ*B signaling. *European Journal of Pharmacology*.

[45] Faubert B., Boily G., Izreig S. (2013). AMPK is a negative regulator of the warburg effect and suppresses tumor growth in vivo. *Cell Metabolism*.

[46] Laderoute K. R., Calaoagan J. M., Chao W. R. (2014). 5′-AMP-activated protein kinase (AMPK) supports the growth of aggressive experimental human breast cancer tumors. *The Journal of Biological Chemistry*.

[47] Smith T. A. D., Zanda M., Fleming I. N. (2013). Hypoxia stimulates 18F-fluorodeoxyglucose uptake in breast cancer cells via hypoxia inducible factor-1 and AMP-activated protein kinase. *Nuclear Medicine and Biology*.

[48] Liu J., Zheng L., Wu N. (2014). Oleanolic acid induces metabolic adaptation in cancer cells by activating the AMP-activated protein kinase pathway. *Journal of Agricultural and Food Chemistry*.

[49] Liu Y., Cao Y., Zhang W. (2012). A small-molecule inhibitor of glucose transporter 1 downregulates glycolysis, induces cell-cycle arrest, and inhibits cancer cell growth in vitro and in vivo. *Molecular Cancer Therapeutics*.

[50] Dando I., Donadelli M., Costanzo C. (2013). Cannabinoids inhibit energetic metabolism and induce AMPK-dependent autophagy in pancreatic cancer cells. *Cell Death and Disease*.

[51] Li Z., Li X., Wu S., Xue M., Chen W. (2014). Long non-coding RNA UCA1 promotes glycolysis by upregulating hexokinase 2 through the mTOR-STAT3/microRNA143 pathway. *Cancer Science*.

